# Left atrial remodeling and mechanical dysfunction in hypertrophic cardiomyopathy

**DOI:** 10.1186/1532-429X-18-S1-P288

**Published:** 2016-01-27

**Authors:** Kyung-Jin Kim, Hong-Mi Choi, Yeonyee E Yoon, Jun-Bean Park, Jin Joo Park, Hyo Eun Park, Seung-Pyo Lee, Goo-Young Cho

**Affiliations:** 1 Division of Cardiology, Cardiovascular Center, Seoul National University Bundang Hospital, Seongnam city, Korea (the Republic of); 2 Healthcare System Gangnam Center, Seoul National University Hospital, Seoul, Korea (the Republic of); 3 Division of Cardiology Department of Internal Medicine, Seoul National University Hospital, Seoul, Korea (the Republic of)

## Background

Patients with hypertrophic cardiomyopathy (HCM) and LA enlargement have more serious cardiovascular events. This study sought to determine the contribution of left atrial (LA) remodeling and mechanical function to hypertrophic cardiomyopathy (HCM) by comparing HCM patients to age- and gender-matched control subjects and young healthy control subjects, and also to assess whether LA remodeling and mechanical function including global strain are related to the characteristics of HCM.

## Methods

A total of 79 patients with HCM who underwent both 2D-speckle tracking echocardiography and cardiac MRI were included (54 ± 12 years; 60 men) and compared to 79 age- and gender-matched control subjects and 20 young healthy control subjects. LA diameter and volume, expansion index for reservoir function, active emptying fraction for pump function, global longitudinal LA strain were measured. The type of HCM, the presence and extent of late gadolinium enhancement (LGE) in left ventricular (LV) myocardium and LV mass index by cardiac magnetic resonance imaging was evaluated.

## Results

When compared to age- and gender-matched control subjects and young healthy control subjects, HCM patients showed an increased LA volume index (30.0 ± 8.7 ml/m^2^ vs 31.3 ± 7.0 ml/m^2^ vs 43.6 ± 15.1 ml/m^2^, *P* value < 0.001), impaired reservoir function (111 ± 42 % vs 150 ± 57 % vs 94 ± 41 %, *P* value < 0.001), decreased LA global strain (27.6 ± 7.7 % vs 31.5 ± 5.6 % vs 22.4 ± 7.9 %, *P* value < 0.001). LA volume index and global strain demonstrated graded association with the extent of LV myocardial scar and LV mass index evaluated by cardiac MRI. (Figure) However, LV mass index was the only independent determinant of LA enlargement and LA global strain rather than LGE extent (LA maximum volume index: ß = 0.358, *P* value = 0.003, LA global strain: ß = -0.295, *P* value = 0.021).

## Conclusions

In HCM, the strongest determinant of LA remodeling and dysfunction is left LV mass index rather than the extent of LV myocardial scar.

**Figure 1:**
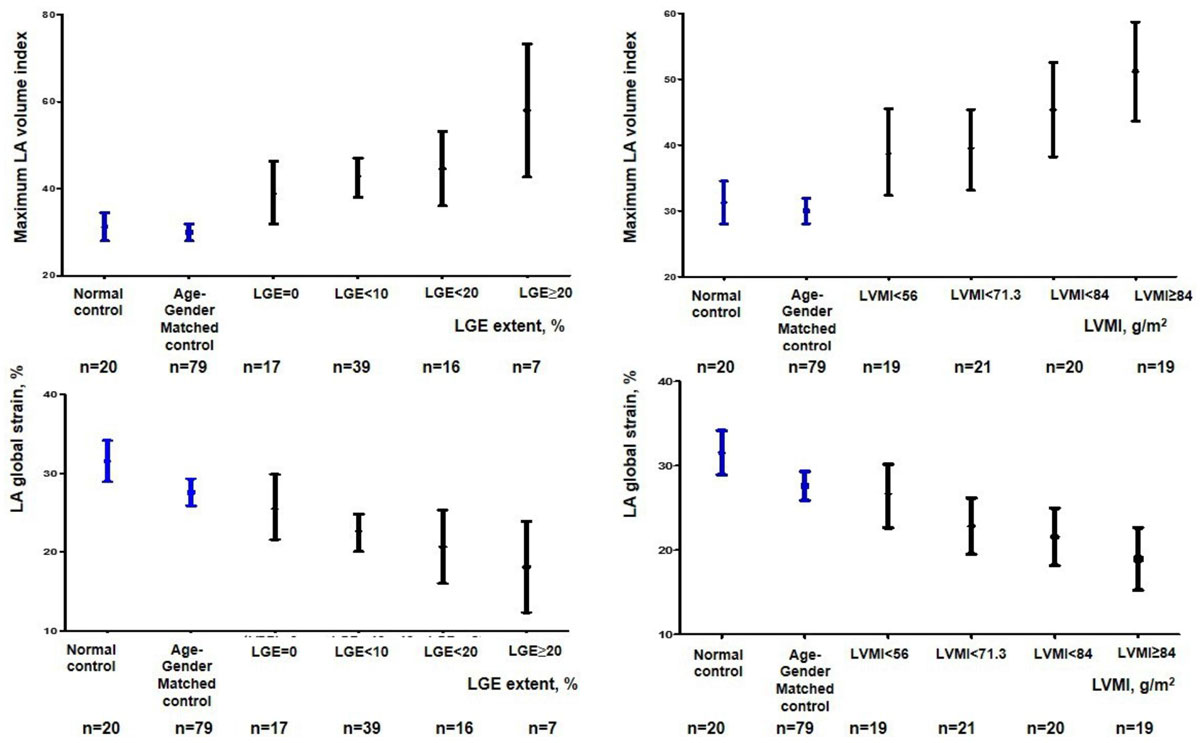
Figure 1

